# Health literacy profile of recently hospitalised patients in the private hospital setting: a cross sectional survey using the Health Literacy Questionnaire (HLQ)

**DOI:** 10.1186/s12913-018-3697-2

**Published:** 2018-11-20

**Authors:** Allison Bourne, Shehzaad Peerbux, Rebecca Jessup, Margaret Staples, Alison Beauchamp, Rachelle Buchbinder

**Affiliations:** 1 Monash Department of Clinical Epidemiology, Cabrini Institute, 4 Drysdale Street, Malvern, VIC Australia; 2 Department of Epidemiology and Preventive Medicine, School of Public Health and Preventive Medicine, Monash University, The Alfred Centre, 99 Commercial Road, Melbourne, VIC Australia; 3 Department of Medicine, Western Health, The University of Melbourne, Sunshine Hospital, Level 3, 176 Furlong Road, WCHRE Building, St Albans, VIC Australia; 4 Australian Institute for Musculoskeletal Science (AIMSS), Sunshine Hospital, Level 3, 176 Furlong Road, WCHRE Building, St Albans, VIC Australia; 5 Health Systems Improvement Unit, Deakin University, 221 Burwood Highway, Burwood, VIC Australia

**Keywords:** Health literacy, Health equity, Quality, Health Literacy Questionnaire, Hospitals

## Abstract

**Background:**

Health service providers should understand and attend to the health literacy needs of their population in view of the known association between low health literacy and poorer health outcomes. This study aimed to determine the health literacy profile of patients treated at a large private hospital in Melbourne, Australia, and any associations between this profile and socio-economic position, health behaviours, health status and use of hospital services.

**Methods:**

A mailed survey was sent to 9173 people aged ≥18 years with a hospital admission between February and October 2014. It included the Health Literacy Questionnaire (HLQ), a multidimensional tool comprising nine independent scales, and socio-demographic and clinical questions. For both respondents and non-respondents, we also extracted residential postcode and admission and follow up details from the Patient Administrative Services database. Differences in demographic, socio-economic and hospital use patterns between respondents and non-respondents were analysed using descriptive statistics. Regression-tests were used to identify differences in health literacy between socio-economic subgroups, with the magnitude of these differences determined using Cohen’s d effect sizes.

**Results:**

There were 3121 respondents (response rate: 35% excluding 154 returned invitations), the majority born in Australia (74.6%) and living in areas of high socio-economic advantage. Respondents were slightly older than non-respondents (mean (SD) age 65.6 (17.0) versus 60.6 (20.8) years) and included proportionately less females (51.9 versus 59.1%) but were similar with regard to other socio-demographic factors and health service use. Participants who did not speak English at home, reported lower scores across several HLQ scales, including those that measure health provider support and engagement. Those who smoked and reported low physical activity had lower scores for actively managing their health. No relationship was seen between HLQ scale scores and use of hospital services.

**Conclusions:**

Based upon the health literacy profile of a large cohort of patients attending a large private hospital, we found no relationship between HLQ scale scores and use of hospital services. However we did identify significant health literacy needs particularly among patients whose primary language at home was not English and patients needing assistance completing the survey. Identifying ways of addressing these needs may improve patient outcomes.

## Background

Adopting mechanisms to measure and address the health literacy needs of patients is becoming an increasingly important component of patient care, particularly in the hospital setting where patients may struggle with navigating the complexities of multi-disciplinary care [1–3]. This change is being driven by the large body of research that has demonstrated the impact of an individual’s health literacy on their health outcomes and use of health care services. For example, low scores on instruments that test functional health literacy (an individual’s ability to read and comprehend health information with or without numeracy skills relating to health information), have been shown to be moderately associated with increased risk of hospital admission [4–6] re-presentation within 30 days [7], and increased emergency department (ED) presentations [6, 8, 9]. Low functional health literacy has also been associated with less use of preventive healthcare services [10], poorer ability to self-manage chronic diseases [11–15], and poorer health outcomes including increased mortality [11, 16–18].

However health literacy is a multifaceted concept that encompasses all aspects related to a person’s ability to find and use health information and navigate the healthcare system [19]. In its broadest sense, it is defined as ‘the cognitive and social skills which determine the motivation and ability of individuals to gain access to, understand and use information in ways which promote and maintain good health’ [20]. This definition indicates the complex nature of health literacy which is influenced by a complex interaction of contextual factors including cultural and personal values, social resources, and individual motivations in addition to their functional health literacy that all influence an individual’s capacity to comprehend and act upon information related to their health.

Advancements in the field have led to the development of new measurement tools that better reflect the wider concept of health literacy, including the European Health Literacy Survey Questionnaire [21], the Swiss Health Literacy Survey [22], the Health Activities Literary Scale [23], the Critical Health Competence Test [24], and the Health Literacy Questionnaire (HLQ) [25]. The HLQ, developed in 2013, includes nine different dimensions of health literacy and its construct validity and reliability have been demonstrated in several contexts [25–28]. The nine independent scales of the HLQ generate a health literacy profile that provides detailed information about an individual’s health literacy skills and deficiencies. Use of the tool within a health service can therefore provide insights into their specific population’s health literacy needs, and help to generate actionable ideas to address them.

We recently reported on the health literacy profile of patients attending a public hospital in a low socio-economic area of Melbourne, Australia [29]. The present study was performed concurrently and its purpose was to better understand the health literacy needs of patients treated at a large private hospital in a high socio-economic area of Melbourne, Australia. The primary aim was to determine the health literacy profile of a large cohort who had been treated as inpatients using the HLQ. A second aim was to determine the associations between an individual’s health literacy, and their socio-economic position, health behaviours and health status, and their use of hospital services.

## Methods

We invited 9173 patients who had been discharged from Cabrini Hospital, Malvern, a 508-bed private not-for-profit acute care hospital to participate in a mailed survey. The hospital is located in southeast Melbourne and delivers emergency, maternity, coronary care, intensive care and paediatric inpatient services. The hospital’s catchment is considered to be a population of advantage, with higher levels of income and educational attainment compared with the State of Victoria’s averages [30].

Data collection took place over 9 months from February to October 2014. Eligible patients were identified through the Patient Administrative Services (PAS) database each month. Patients were eligible to receive a survey if they had been admitted for at least 24 h in the past 30 days, aged 18 years or over, and not discharged with a diagnosis of dementia or to palliative or hospice care. In the first month, a computer generated random sample of 50 patients were sent surveys to pilot test the process. This was followed by 100 patients in the second month. From April to September, all hospitalised individuals who met the eligibility requirements were sent surveys. Overall, a total of 9173 surveys were sent.

To maximise response rates, eligible recently discharged patients were sent a pre-notification letter informing them about the survey. One week later, they were sent a personalised cover letter instructing them about the purpose of the study, the information and consent form, the survey and a reply-paid envelope. Non-respondents were sent a reminder 3 weeks after the first survey was sent. Letters explained that the survey was being conducted to help improve the standard of care at the hospital and were co-signed by the hospital’s Chief Executive Officer and the Principal Investigator (RB). They also encouraged participants to complete the survey themselves, or with help if needed. All participants were allocated a unique study ID. No information from this study was included in the patients’ medical record. Reasons for non-participation were recorded where known.

### Measures

#### Health literacy questionnaire (HLQ)

The HLQ has 44 items covering nine domains of health literacy captured in nine independent scales*: 1. Feeling understood and supported by healthcare providers, 2. Having sufficient information to manage my health, 3. Actively managing my health, 4. Social support for health, 5. Appraisal of health information, 6. Ability to actively engage with healthcare providers, 7. Navigating the healthcare system, 8. Ability to find good health information,* and *9. Understanding health information well enough to know what to do.*

Each scale consists of between four and six items. The first five scales are scored from 1 (*strongly disagree*) to 4 (*strongly agree*). The last four scales scored from 1 (*cannot do* or *always difficult*) to 5 (*very easy*). Scales are scored independently by adding the scores for each item within the scale, and dividing by the number of items within the scale.

#### Socio-demographic, health and hospital use variables

Self-reported socio-demographic data were collected for age, sex, height, weight, living arrangements, Aboriginal and Torres Strait Islander status, country of birth, whether or not English was the primary language spoken at home, education level, work status, internet usage, private health insurance status, household income, government benefits (if applicable), alcohol consumption, smoking status, physical activity levels, pre-existing health conditions, self-reported Emergency Department (ED) presentations in the last 12 months, and whether or not the participant had help completing the survey.

For both respondents and non-respondents, we extracted data from the PAS database including admission source, admission type, discharge destination, hospital length of stay, ED presentations and hospital admissions to any Cabrini Health acute hospital facility (Malvern or Brighton) in the 12 months following the index admission. Residential postcode was also extracted to determine Index of Relative Socio-economic Disadvantage (IRSD) status related to home address. This index is produced by the Australian Bureau of Statistics as part of the Socio-Economic Index for Areas (SEIFA) and ranks areas in Australia into deciles using census data according to relative socio-economic advantage and disadvantage [31]. Scores range from 1 to 10 with lower scores indicating more disadvantage than other areas of Australia.

### Ethical considerations

Ethical approval was obtained from both the Cabrini Human Research Ethics Committee (EC00239) and the Monash University Human Research Ethics Committee (EC00234). In order to avoid potential harm or embarrassment to participants, ensure unbiased response, and maximise the response rate, the term ‘literacy’ was not used in communications with participants. A previous qualitative study of low literacy individuals found that the expression discourages individuals with low literacy from engaging in healthcare experiences and results in persistent anxiety regarding their literacy difficulties [32]. As this was a study assessing patient’s (health) literacy, participants were encouraged to complete the documents with assistance if necessary to ensure that all participants, including those with impaired literacy, clearly understood the study and could therefore provide fully informed consent.

All respondents provided written informed consent. We also requested consent to access respondents’ hospital records. Where consent was obtained we extracted re-identifiable information. For respondents who did not provide consent for their hospital records to be reviewed, and for non-respondents, only non-identifiable data were extracted. Consent for de-identified data for respondents who did not consent for their hospital records to be reviewed, and for non-respondents was deemed unnecessary by the Cabrini Human Research Ethics Committee.

### Statistical analysis

Data were analysed using Stata 13 (Stata Corp). Missing values for the HLQ were imputed as described by Beauchamp et al. [33]. To determine whether survey respondents differed from non-respondents, Kruskall Wallis or t-tests, depending on data type and distributional characteristics, were used to examine differences in demographic, socio-economic and hospital use patterns between respondents and non-respondents.

For respondents who agreed to medical record access, Pearson chi-square tests were used to determine whether there were associations between health literacy and demographic, socio-economic, and hospital use variables. Where differences were found, Cohen’s d effect sizes were calculated to describe the magnitude of differences in HLQ scores and interpreted as a ‘small’ effect size if Cohen’s d was > 0.20–0.50, ‘medium’ effect size if 0.50–0.80, and ‘large’ effect size for results > 0.80 [34].

## Results

**Fig. 1:**
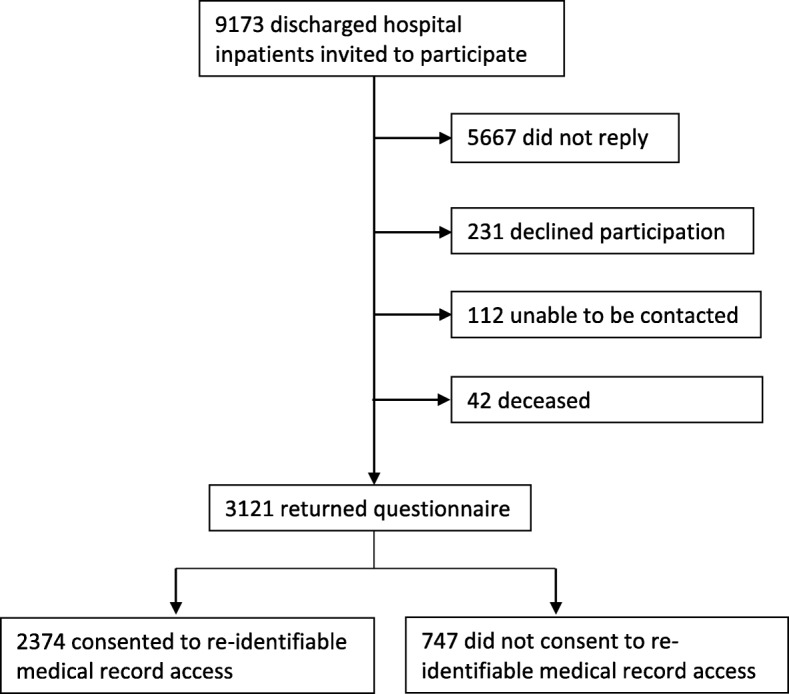
Recruitment process for health literacy cross sectional survey

A total of 9173 surveys were sent (refer to Fig. 1). Of these, 154 were returned as the patient had died or the address was incorrect. A total of 3118 patients responded to the survey (response rate excluding non-contactable patients was 35%) and of these, 2374 (76%) consented to review of their medical records.

Table 1 compares respondents and non-respondents. Respondents were older (mean age 65.6 versus 60.6 years), and proportionately less females responded (51.9% versus 59.1%). There were no significant differences between respondents and non-respondents with regard to language spoken at home, IRSD deciles (9 IQR 8, 10), length of stay, or ED presentations in the 12 months following the index hospital admission, but more respondents were readmitted to hospital in the 12 months following the index admission (37.9% versus 32.6%).

**Table 1:** Demographic and hospital use data of respondents versus non-respondents where available

Variables	Respondents *N* = 3118	Non-respondents *N* = 6055	*P*-value^+^
	N (%)	N (%)	
Age, years, mean (SD)	65.6 (17.0)	60.6 (20.8)	< 0.001
Female	1618 (51.9)	3577 (59.1)	< 0.001
Born in Australia	2325 (74.6)	4075 (67.3)	< 0.001
IRSD^a^, median (IQR)	9 (8, 10)	9 (8, 10)	0.17
Length of stay, days, median (IQR)	3 (1, 5)	3 (1, 5)	0.09
Admission Type			< 0.001
Planned admission	1756 (56.6)	3003 (49.6)	
Emergency Department (ED)	905 (29.0)	2026 (33.5)	
Unscheduled community	230 (7.4)	438 (7.2)	
Maternity	218 (7.0)	588 (9.7)	
Admitting specialty			< 0.001
Cardiology	528 (16.9)	773 (12.8)	
Obstetrics/Gynaecology	321 (10.3)	804 (13.3)	
Gastroenterology	375 (12.0)	738 (12.2)	
Urology	285 (9.1)	511 (8.4)	
Neurology	228 (7.3)	398 (6.6)	
^b^Surgery other	597 (19.1)	1205 (19.9)	
^c^Medical other	577 (18.5)	1236 (20.4)	
Hospital readmission within 12 months	1181 (37.9)	1974 (32.6)	< 0.001
Cabrini ED attendance within 12 months	605 (19.4)	1161 (19.2)	0.46

^+^*P*-value calculated using Kruskall Wallis or t-tests, depending on data type and distributional characteristics

^a^*IRSD* Index of relative socio-economic disadvantage deciles based on postcodes

^b^Surgery other includes orthopaedics, plastic, general, vascular and ENT

^c^Medical other includes general medicine, oncology/haematology, hospital in the home, respiratory, endocrinology, nephrology and geriatric medicine

More than half (52%) of respondents were retired and most (86%), were on an annual income greater than $30,000 (Table 2). Most (78%) had at least one longstanding illness or disability, and 12% had four or more chronic conditions. Almost half (47%) had completed a tertiary education qualification and nearly all (95%) had private health insurance. Eight percent (*N* = 242) required assistance in completing the survey due to either physical disability or difficulties with comprehension. Study population mean scores for each HLQ scale are shown in Table 3. The lowest score was reported for ‘*Appraisal of health information’* (mean (SD): 2.85 (0.53), score range 1 to 4) and the highest score was reported for ‘*Understanding health information well enough to know what to do’* (mean (SD) 4.11 (0.58), score range 1 to 5).

**Table 2:** Self-reported demographic, health and hospital usage characteristics of respondents to the survey, *N* = 3118^a^

Demographic, health and hospital characteristics	
	Median (IQR)
Body Mass Index (BMI), kg/cm^2^ (*N* = 2782)	25.8 (23.1, 29.0)
	N (%)
Lives alone (*N* = 1276)	635 (20.6)
Aboriginal/Torres Strait Islander (*N* = 3092)	6 (0.2)
English speaking at home (*N* = 3098)	2992 (96.7)
Highest level of education (*N* = 3070)	
Did not complete secondary education	564 (18.3)
High school completed	596 (19.4)
TAFE or Trade completed	477 (15.5)
Completed undergraduate university degree	934 (30.4)
Completed postgraduate university degree	499 (16.3)
Employment Status (*N* = 3075)	
Retired	1610 (52.4)
Working part/full-time	988 (32.1)
Home duties	217 (7.1)
Permanently unable to work/ill	79 (2.6)
Student	41 (1.3)
Other employment status^b^	140 (4.6)
In receipt of benefits (age, Veterans Affairs, unemployment, disability or other pension (*N* = 3073)	1033 (33.1)
Household annual income (*N* = 3118)	
Less than $30,000	413 (13.2)
$30,000–$49,999	426 (13.7)
$50,000–$74,999	379 (12.2)
$75,000–$99,999	303 (9.7%)
≥ $100,000	789 (25.3)
Rather not say/did not indicate	808 (25.9%)
Current smoker (*N* = 3065)	98 (3.2)
Alcohol consumption (*N* = 3065)	
Never	508 (16.0)
Sometimes	1627 (53.1)
2 or less standard glasses a day	713 (23.3)
More than 2 standard glasses a day	217 (7.1)
Internet usage (*N* = 3069)	
Never/Less than once a month	583 (19.0)
At least once a month or more	2486 (80.9)
Longstanding illness or disability (*N* = 3056)	
≥ 4 chronic conditions	358 (11.5)
Arthritis	951 (31.1)
Back Pain	817 (26.7)
Heart Problems	916 (30.0)
Asthma or lung condition	462 (15.1)
Cancer	520 (117.2)
Depression or anxiety	396 (13.0)
Diabetes	353 (11.6)
Stroke	122 (4.0)
Other conditions	685 (22.4)
None	674 (22.1)
Private health insurance (*N* = 3075)	2918 (94.9)
Physical activity ≥3.5 h exercise/week (*N* = 1285)	2210 (70.8)
Self-reported ED admission in the last 12 months (*N* = 3090)	1653 (53.5)
Needed assistance to complete the questionnaire (*N* = 3094)	242 (7.8)

^a^Not all respondents provided data for all questions in the survey

^b^Other employment included temporarily unable to work, causal employment, unemployed or maternity leave

**Table 3:** Mean and standard deviation of the nine HLQ scales for patients recently hospitalised in a private hospital, *N* = 3121

HLQ scales^a^	Mean (SD)
1. Feeling understood and supported by healthcare providers	3.35 (0.48)
2. Having sufficient information to manage health	3.07 (0.48)
3. Actively managing health	3.07 (0.49)
4. Social support for health	3.26 (0.49)
5. Appraisal of health information	2.85 (0.53)
6. Ability to actively engage with healthcare providers	4.07 (0.58)
7. Navigating the healthcare system	3.91 (0.57)
8. Ability to find good health information	3.85 (0.62)
9. Understanding health information well enough to know what to do	4.11 (0.58)

^a^Score range for scales 1 to 5 is 1 to 4, with higher scores indicating greater agreement, and score range for scales 6 to 9 is 1 to 5, with higher scores indicating less difficulty

Table 4 displays the socio-demographic, health and hospital use variables and their association with the nine scales of the HLQ. The largest effect size for difference in means was found for those that needed assistance with completing the survey, with large effect sizes found for those requiring assistance compared to those not requiring assistance for the scales ‘*Finding health information’* and ‘*Understanding health information well enough to know what to do*;’, and small effect sizes found for ‘*Actively managing my health’*, ‘*Appraisal of health information’*, and ‘*Social support for health*’. Speaking a language other than English at home was associated with the largest number of health literacy scales, with those who did not speak English at home having significantly lower scores (small to medium effect sizes), than those who spoke English, for the scales ‘*Health care provider support’*, ‘*Having sufficient information’*, ‘*Active engagement with healthcare professionals’*, ‘*Ability to find good health information’*, and ‘*Understanding health information well enough to know what to do’*.

**Table 4:** Association between HLQ scores, demographic health and hospital use subgroups (*N* = 2374)

		Healthcare provider support	Having sufficient information	Actively managing health	Social support for health	Active appraisal of health information	Active engagement with healthcare professionals	Navigating the healthcare system	Ability to find good health information	Understanding health information
	Variable (*N*)	Mean (SD)	Mean (SD)	Mean (SD)	Mean (SD)	Mean (SD)	Mean (SD)	Mean (SD)	Mean (SD)	Mean (SD)
Sex	Female *(1604)*	3.34 (0.48)	3.12* (0.48)	3.12 (0.48)	3.25 (0.50)	2.92* (0.53)	4.05 (0.60)	3.91 (0.58)	3.90 (0.63)	4.15 (0.58)
	Male (*1487*)	3.35 (0.48)	3.02* (0.47)	3.03 (0.49)	3.26 (0.48)	2.77* (0.52)	4.09 (0.55)	3.90 (0.55)	3.79 (0.61)	4.07 (0.57)
Age	< 65 years (*1216*)	3.33 (0.50)	3.10 (0.48)	3.09 (0.51)	3.24 (0.48)	2.90 (0.52)	4.05 (0.57)	3.89 (0.56)	3.95 (0.57)	4.17 (0.54)
	≥ 65 years (*1853*)	3.36 (0.47)	3.06 (0.48)	3.07 (0.48)	3.27 (0.49)	2.81 (0.53)	4.08 (0.58)	3.93 (0.56)	3.78 (0.64)	4.08 (0.60)
BMI	BMI ≤ 25 (*1182*)	3.34 (0.49)	3.09 (0.48)	3.13 (0.50)	3.28 (0.47)	2.84 (0.53)	4.06 (0.59)	3.89 (0.56)	3.85 (0.62)	4.13 (0.58)
	BMI > 25 (*1582*)	3.36 (0.47)	3.07 (0.47)	3.04 (0.49)	3.25 (0.49)	2.86 (0.53)	4.09 (0.56)	3.92 (0.56)	3.87 (0.61)	4.12 (0.56)
Lives alone	Yes *(630*)	3.35 (0.48)	3.04 (0.49)	3.09 (0.48)	3.13* (0.56)	2.80 (0.54)	4.06 (0.62)	3.90 (0.57)	3.77 (0.68)	4.09 (0.62)
	No (*2421*)	3.32 (0.49)	3.08 (0.48)	3.07 (0.49)	3.29* (0.46)	2.86 (0.53)	4.07 (0.57)	3.91 (0.56)	3.87 (0.60)	4.12 (0.56)
Born in Australia	Yes (*2308*)	3.37 (0.48)	3.09 (0.47)	3.09 (0.49)	3.27 (0.48)	2.85 (0.53)	4.09 (0.57)	3.93 (0.56)	3.86 (0.61)	4.13 (0.55)
	No (*781*)	3.28 (0.49)	3.03 (0.51)	3.04 (0.49)	3.22 (0.50)	2.84 (0.52)	4.02 (0.61)	3.85 (0.58)	3.80 (0.65)	4.07 (0.64)
English spoken at home	Yes (*2967*)	**3.35 (0.48)**	3.08* (0.48)	3.08 (0.49)	3.26 (0.49)	2.85 (0.53)	4.08* (0.57)	3.92* (0.56)	3.86** (0.62)	4.13** (0.56)
	No (*98*)	**3.23 (0.52)**	2.95* (0.49)	2.99 (0.47)	3.21 (0.50)	2.85 (0.51)	3.83* (0.70)	3.67* (0.64)	3.58** (0.73)	3.76** (0.86)
Employed	Yes (*1121*)	3.35 (0.49)	3.11 (0.48)	3.10 (0.51)	3.24 (0.48)	2.89 (0.53)	4.08 (0.57)	3.91 (0.56)	3.95* (0.57)	4.18 (0.53)
	No (*1927*)	3.35 (0.47)	3.06 (0.47)	3.06 (0.47)	3.27 (0.49)	2.82 (0.53)	4.07 (0.58)	3.91 (0.57)	3.78* (0.64)	4.07 (0.60)
Left school/TAFE^a^ before completion	Yes (*1637*)	3.31 (0.49)	3.03 (0.49)	3.03 (0.49)	3.24 (0.50)	2.77* (0.53)	4.04 (0.61)	3.89 (0.58)	3.73* (0.66)	4.00* (0.66)
	No (*1485*)	3.36 (0.47)	3.09 (0.47)	3.10 (0.49)	3.27 (0.48)	2.88* (0.53)	4.08 (0.56)	3.92 (0.56)	3.90* (0.59)	4.17* (0.54)
Receives government benefits	Yes (*1024*)	3.36 (0.46)	3.06 (0.47)	3.06 (0.48)	3.26 (0.50)	2.80 (0.53)	4.07 (0.59)	3.92 (0.57)	3.74* (0.65)	4.03* (0.62)
	No (*2068*)	3.34 (0.49)	3.08 (0.48)	3.08 (0.49)	3.26 (0.48)	2.87 (0.53)	4.07 (0.57)	3.91 (0.56)	3.90* (0.60)	4.15* (0.55)
Household income <$30,000 p.a.	Yes (*1884*)	3.37 (0.48)	3.09 (0.47)	3.09 (0.50)	3.27 (0.47)	2.87 (0.53)	4.10* (0.56)	3.93* (0.56)	3.90* (0.60)	4.16* (0.54)
	No (*412*)	3.30 (0.47)	3.02 (0.46)	3.04 (0.48)	3.17 (0.55)	2.78 (0.53)	3.94* (0.64)	3.80* (0.58)	3.63* (0.67)	3.94* (0.66)
Internet use < once a month	Yes (*583*)	3.30 (0.47)	3.03 (0.48)	3.02 (0.48)	3.26 (0.51)	2.71* (0.53)	4.01 (0.63)	3.87 (0.61)	3.61* (0.75)	3.93* (0.68)
	No (*2486*)	3.36 (0.48)	3.08 (0.48)	3.09 (0.49)	3.26 (0.48)	2.88* (0.53)	4.08 (0.56)	3.92 (0.56)	3.90* (0.57)	4.16* (0.54)
Private health insurance	Yes (*2892*)	3.35 (0.48)	3.07 (0.47)	3.08 (0.49)	3.26 (0.48)	2.85* (0.53)	4.07 (0.57)	3.91 (0.56)	3.85 (0.62)	4.12 (0.57)
	No (*156*)	3.33 (0.48)	3.06 (0.54)	3.03 (0.54)	3.22 (0.56)	2.71* (0.62)	4.05 (0.68)	3.96 (0.62)	3.78 (0.72)	4.04 (0.64)
Physical activity ≥3.5 h per week	Yes (*2192*)	3.36 (0.48)	3.09 (0.47)	3.13* (0.48)	3.27 (0.48)	2.87 (0.53)	4.08 (0.57)	3.92 (0.57)	3.87 (0.61)	4.13 (0.56)
	No (*898*)	3.31 (0.48)	3.04 (0.50)	2.94* (0.49)	3.23 (0.50)	2.80 (0.54)	4.04 (0.60)	3.88 (0.56)	3.80 (0.65)	4.07 (0.61)
≥ 4 health conditions	Yes (*354*)	3.40 (0.46)	3.00 (0.51)	3.02 (0.48)	3.21 (0.55)	2.85 (0.51)	3.99 (0.59)	3.84 (0.60)	3.73* (0.68)	3.99* (0.63)
	No (*2737*)	3.34 (0.48)	3.08 (0.47)	3.08 (0.49)	3.26 (0.48)	2.85 (0.53)	4.08 (0.58)	3.92 (0.56)	3.86* (0.61)	4.13* (0.57)
Current smoker	Yes (*96*)	3.25 (0.55)	3.00 (0.52)	2.89* (0.54)	3.12* (0.63)	2.75 (0.52)	3.98 (0.69)	3.82 (0.63)	3.68 (0.73)	4.06 (0.57)
	No (*2941*)	3.35 (0.48)	3.08 (0.47)	3.08* (0.49)	3.26* (0.48)	2.85 (0.53)	4.07 (0.57)	3.91 (0.56)	3.85 (0.62)	4.11 (0.58)
Alcohol consumption	Yes (*2537*)	3.35 (0.48)	3.08 (0.47)	3.07 (0.49)	3.26 (0.48)	2.85 (0.53)	4.07 (0.55)	3.91 (0.55)	3.86 (0.60)	4.12 (0.55)
	No (*500*)	3.35 (0.47)	3.06 (0.50)	3.10 (0.48)	3.26 (0.51)	2.83 (0.54)	4.06 (0.68)	3.91 (0.63)	3.79 (0.72)	4.09 (0.70)
Needed help with questionnaire	Yes (*239*)	3.33 (0.47)	3.00 (0.51)	2.97* (0.53)	3.37* (0.45)	**2.64 (0.57)**	3.86* (0.68)	3.73 (0.63)	3.40*** (0.77)	3.62*** (0.79)
	No (*2825*)	3.35 (0.48)	3.08 (0.47)	3.08* (0.49)	3.25* (0.49)	**2.86 (0.53)**	4.09* (0.56)	3.93 (0.56)	3.88*** (0.59)	4.16*** (0.53)
Self-reported ED admission	Yes (*1632*)	3.35 (0.49)	3.05 (0.49)	3.06 (0.50)	3.26 (0.50)	2.81 (0.54)	4.05 (0.59)	3.89 (0.57)	3.80 (0.65)	4.09 (0.61)
	No (*1429*)	3.34 (0.47)	3.10 (0.46)	3.09 (0.48)	3.25 (0.48)	2.89 (0.52)	4.09 (0.56)	3.93 (0.56)	3.90 (0.59)	4.15 (0.54)
Re-presentation to ED within 12 months	Yes (*596*)	3.34 (0.49)	3.05 (0.51)	3.06 (0.51)	3.29 (0.49)	2.83 (0.54)	4.06 (0.62)	3.90 (0.60)	3.76 (0.70)	4.07 (0.62)
	No (*2495*)	3.35 (0.48)	3.08 (0.47)	3.08 (0.48)	3.25 (0.49)	2.86 (0.52)	4.07 (0.57)	3.91 (0.56)	3.87 (0.60)	4.12 (0.56)
Re-admission to hospital	Yes (*1164*)	3.36 (0.49)	3.07 (0.49)	3.08 (0.50)	3.29 (0.50)	2.83 (0.54)	4.08 (0.59)	3.91 (0.57)	3.78 (0.65)	4.08 (0.59)
	No (*1927*)	3.34 (0.48)	3.08 (0.47)	3.07 (0.49)	3.23 (0.48)	2.86 (0.52)	4.06 (0.57)	3.91 (0.56)	3.89 (0.60)	4.13 (0.57)

Bolded results indicate where there were differences in mean HLQ scale scores between individual demographic, socio-economic or hospital use variables according to Pearson chi-square tests, *p* < 0.05. The interpretation of the size of the differences in means was determined using Cohen’s d for difference in means where a Cohen’s d of > 0.20–0.50 indicated a small effect size*, > 0.50–0.80 indicated a medium effect size** and > 0.80 indicated a large effect size***

^a^Technical and Further Education

The scales where the largest number of differences in subgroups across socio-demographic variables were seen were ‘*Ability to find good health information’* and ‘*Understanding Health information well enough to know what to do’*. Not speaking English at home, being unemployed, not having a tertiary qualification, receiving government benefits, having a household income less than $30,000, using the internet less than daily, having four or more chronic health conditions, and requiring assistance with the survey were all associated with lower reported scores for ‘*Ability to find good health information*’. With the exception of employment status, these variables also resulted in lower scores for ‘*Understanding health information well enough to know what to do’*.

Current smokers and/or individuals who recorded doing less than 3.5 h of physical activity per week were more likely to report lower scores for ‘*Actively managing health’* compared with non-smokers and physically active individuals. Current smokers, as well as those who live alone, were also more likely to report lower ‘*Social support for health’* compared with non-smokers and those who lived with others respectively. We found no relationship between HLQ scale scores and length of stay or further hospital admissions and ED presentations in the year following the index admission.

## Discussion

This study describes the health literacy profile of a large cohort of patients discharged from a large private hospital in a high socio-economic area of metropolitan Melbourne. Despite living in areas of significant socio-economic advantage and high education attainment, we identified significant health literacy needs in this patient population. In particular, respondents who spoke a primary language other than English at home, and those who needed help to complete the survey, were found to have the largest number of low scores across HLQ scales. However, there was no association between greater use of hospital services and lower health literacy scores across any of the nine HLQ scales.

Our study agrees in part with previous studies that have found that socio-economic factors (low educational attainment and low income), are associated with lower levels of health literacy as measured by functional health literacy tools [35–37]. We found that respondents who were unemployed, with very low household incomes, and those without a completed tertiary qualification, to have significantly lower scores across some health literacy scales. However, we also found that poor health behaviours (low levels of physical activity and smoking), using the internet less than once a day, living alone, speaking a language other than English, requiring assistance to fill in the survey, and being male, were all also associated with lower scores in some scales on the health literacy questionnaire.

The strong association between speaking a primary language other than English at home and having lower scores across a number of health literacy scales has been described in previous studies [33, 36, 38]. Our research provides insight into areas of health literacy difficulty that go beyond poor reading comprehension and numeracy skills for health-related materials, and identifies that these individuals experience challenges navigating the healthcare system, and in feeling meaningfully engaged with healthcare providers. Population-based surveys have found that an individual’s health care provider is their most frequently used and most trusted source of health information [39–41]. Poor or no relationships with health care providers could therefore impact significantly on an individual’s access to information about their condition and their ability to manage it effectively. This suggests that ensuring that all patients with non-English backgrounds have strong relationships with at least one health care provider, and strategies to improve the navigability of the health care system, are likely to be important ways of addressing their health literacy needs.

While our finding of no association between lower health literacy scores and increased use of hospital services is in agreement with findings from a previous survey using the HLQ in a cohort of Australian public hospital patients [29], it contrasts with much of the previous research [4–8, 42]. There are a number of possible explanations for these discrepancies. Most of the earlier studies have used functional health literacy instruments to investigate any association between hospital service utilisation and health literacy. Our use of a multidimensional instrument that measures ability and needs separately across the different scales demonstrate that self-reported strengths in some aspects of health literacy may compensate for deficiencies in other scales. For example, it is possible that good social support like a partner with high health literacy skills, and/or strong engagement with health professionals, may compensate for an individual’s poor reading comprehension skills, resulting in sufficient capacity to self-manage without an increased need of health services. We also found that Individuals requiring assistance to fill in the survey reported greater levels of social support for health, but reported feeling less engaged with health care providers. This suggests that social support for health may be critical for helping people with low literacy skills access and engage with the healthcare system.

Strengths of our study include the very large sample size and minimal differences between respondents and non-respondents, indicating that our results are likely to be generalisable to the private hospital’s patient population. On the other hand, while we tried to reduce the risk of a biased response by not mentioning literacy in the survey invitation, those with low literacy and/or health literacy may have been less inclined to participate, which may have resulted in an overestimate of the health literacy of this population. Further, those who spoke a language other than English at home were not provided surveys in their language of origin, and this may have also influenced the way the surveys were filled out, as well as the response rate in patient group.

The early conceptualisation of health literacy as an individual’s ability to read and comprehend written health-related materials has led to a belief that it is a relatively unmodifiable ‘condition’. Most interventions directed to people with lower levels of health literacy have therefore focused on strategies that compensate for, rather than improve health literacy (such as simplifying written health information) [16, 43]. Recently, research has identified the attributes of organisations that provide services which respond to individuals with different health literacy needs [2, 44]. Future research should now focus on determining whether development of these organisation attributes, in combination with targeted approaches to address the specific health learning needs of the individual (in the context of each individual’s learning style, language and preferences), lead to improvements in patient and carer health literacy.

## Conclusions

Our study has determined the health literacy profile of a large cohort of patients attending Cabrini Hospital, a large private not-for-profit hospital in Melbourne, Australia. Despite high socio-economic backgrounds, we found that those who did not speak English at home, those who required assistance completing the survey, those who lived alone, those who do not use the internet each day, and those presenting with poorer health behaviours (smoking and low physical activity), exhibited specific health literacy needs. We plan to present these data to Cabrini Health stakeholders (patients, staff and management) to elicit ideas for how the hospital can address these health literacy needs. For example the HLQ or selected scales could be included in the standard pre-admission form, health literacy could be integrated into pathway planning, and relevant staff may be required to complete a mandatory health literacy e-learning module.

## Abbreviations

ED: Emergency Department
HLQ: Health Literacy Questionnaire
IRSD: Index of Relative Socio-economic Disadvantage
PAS: Patient Administrative Services
